# Retraction: Do natural environmental protection, regional innovation climate, entrepreneurs’ cognition of green development positively influence the sustainable development of small rural businesses

**DOI:** 10.1371/journal.pone.0348046

**Published:** 2026-04-27

**Authors:** The PLOS One Editors

Following the publication of this article [1], concerns were raised about:

- High similarity with results reported in [2], including identical values reported for the overall number of respondents and population attributes reported in Table 1 and S1 File of [1] and Table 2 of [2] (number of respondents per region, position, number of employees, annual turnover, age).
- Compliance with PLOS policy on Authorship.

The corresponding author stated that the two articles [1,2] report results from separate and distinct online surveys that were conducted at different times. The Editors do not consider this information to be sufficient to address the concern regarding high similarity between the study populations.

The *PLOS One* Editors retract this article due to concerns about the reliability and integrity of the reported results.

XZ did not agree with the retraction. JT and AK either did not respond directly or could not be reached.
